# Phase separation in supramolecular and covalent adaptable networks

**DOI:** 10.1039/d3sm00047h

**Published:** 2023-04-03

**Authors:** Martijn H. P. de Heer Kloots, Sybren K. Schoustra, Joshua A. Dijksman, Maarten M. J. Smulders

**Affiliations:** a Laboratory of Organic Chemistry, Wageningen University Stippeneng 4 6708 WE Wageningen The Netherlands maarten.smulders@wur.nl; b Physical Chemistry and Soft Matter, Wageningen University Stippeneng 4 6708 WE Wageningen The Netherlands; c Van der Waals-Zeeman Institute, Institute of Physics, University of Amsterdam Science Park 904 1098 XH Amsterdam The Netherlands j.a.dijksman@uva.nl

## Abstract

Phase separation phenomena have been studied widely in the field of polymer science, and were recently also reported for dynamic polymer networks (DPNs). The mechanisms of phase separation in dynamic polymer networks are of particular interest as the reversible nature of the network can participate in the structuring of the micro- and macroscale domains. In this review, we highlight the underlying mechanisms of phase separation in dynamic polymer networks, distinguishing between supramolecular polymer networks and covalent adaptable networks (CANs). Also, we address the synergistic effects between phase separation and reversible bond exchange. We furthermore discuss the effects of phase separation on the material properties, and how this knowledge can be used to enhance and tune material properties.

## Abstract 2

In this review we highlight the underlying mechanisms of phase separation in dynamic polymer networks, distinguishing between supramolecular networks and covalent adaptable networks, and discuss the effect of phase separation on material properties.

## Graphical abstract

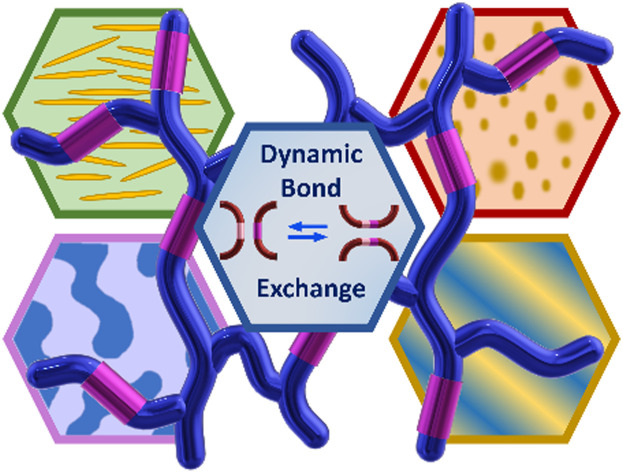

## 1 Introduction

Phase separation is a well known phenomenon in polymer materials that strongly affects the physical properties of the material. Phase separation typically occurs when non-ideal (repulsive) interactions manifest within an inhomogeneous mixture. In such a system, beyond a certain threshold concentration, a demixing process sets in. The system gives up some mixing entropy while unfavourable interactions – the enthalpic contributions – are reduced, such that the (Gibbs) free energy is minimised. At equilibrium, this should lead to two macroscopic coexisting phases, separated by a single interface. In macromolecular systems, which are characterised by relatively little mixing entropy, phase separation can already be found for weak monomer-level driving forces.^1^ This causes phase separation processes to be very common compared to its occurrence in low molecular weight analogues. However, the complete equilibration of such phase-separating systems is, especially for long polymers, not a given; the system may end up in a kinetically trapped state. Such composite materials may then feature separated regions (phases) of a preferentially tuneable length scale with different local concentrations and clear borders.^2^

An interesting strategy in materials science is to use these kinetically trapped states for structuring and imparting new functionalities within polymeric materials. In macromolecular (often polymeric) systems, phase separation is frequently applied to enhance mechanical strength, to facilitate order on a wide range of different length scales, or to add localised functionalities to the material.^3,4^ Prominent examples of the use of phase separation in polymer networks include the formation of *meso* structures in (porous) membranes,^5^ solar cells and batteries,^6^ improved mechanical performance in polyurethanes,^7,8^ and in colloidal gels.^9^

A wide range of possible phase-separating materials exists and, hence, there is a high variability in the relevant length scales. Also, phase separation is not restricted to man-made systems. Indeed phase separation in bio-polymers, such as proteins, is also commonly found in nature.^10^ Phase-separating materials are, for example, used to provide structure to cells,^11^ but they are also found on larger length scales where polymer materials provide structural colours in, *e.g.*, bird feathers.^12^ Such examples from nature in turn have sparked increased interest in the application of phase separation to add new and specific functionalities in synthetic materials. Conversely, physical–chemical understanding of phase separation (*e.g.*, by modelling and experimentation), can also serve as a means to better understand phase separation in biological systems.^13^

For (synthetic) polymer systems, phase separation is typically divided into two classes: liquid–liquid phase separation (LLPS) and solid–liquid phase separation (SLPS).^14^ For dynamic polymer networks, however, the boundary between these two phases is not always easy to identify; after all, many of these materials are viscoelastic. Therefore, instead of focusing on the division into these two categories, we will discuss phase separation holistically. Several books and reviews have elaborated further on the deeper mechanisms and concepts of phase separation, such as the chapter on Polymer phase separation in Polymer Physics by Hu,^2^ or the excellent reviews for phase separation in thin polymer films,^15^ in membrane formation^16^ and due to reactions in polymer blends.^17^ However, in this review we will only address the basic concepts that will aid in the later discussions on phase separation in dynamic polymer networks.

### 1.1 Describing phase separation in polymeric systems

A starting point for describing phase separation of polymer mixtures is the well-known Flory–Huggins interaction parameter χ.^18,19^ This parameter expresses the balance between (1) the self-interactions of polymer molecules, (2) the interactions between solvent molecules and (3) interactions between polymer and solvent molecules. This balance is critical in defining the enthalpic contributions to the Gibbs energy of mixing.

**Fig. 1:**
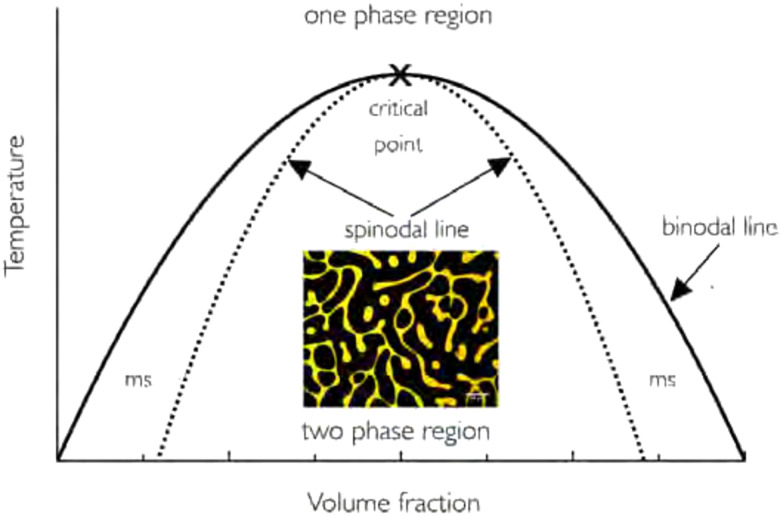
Example of a phase diagram for a polymer–solvent system. The binodal line separates the one-phase region with the phase-separated two-phase region. The metastable (ms) region lies between the binodal and spinodal regions. The included image corresponds to spinodal decomposition of the system. Note that in reality, for most polymer solutions, the phase diagram is often asymmetric. Reprinted with permission from ref. 12 Copyright 2018 IOP Publishing Ltd.

From the Gibbs energy considerations, conclusions on (phase) behaviour at different compositions follow. Phase diagrams are often constructed in which the composition of a solution is plotted against a control variable, such as the temperature (in Flory–Huggins theory often χ) (Fig. 1). Within these diagrams, the binodal and spinodal curves define the kinetic route the system will follow upon phase separation. Within the spinodal, infinitesimally small fluctuations in temperature or density will lead to phase separation. Between the binodal and spinodal, a long-lived metastable state is possible, in which the two substances occur in a mixed phase. Phase separation can be induced, but only occurs *via* nucleation and growth. Both of these mechanisms are relevant for polymeric mixtures, yet more intricacies may present themselves during network formation, such as changes in size and interactions of species due to reactions (*e.g.*, in polymerising mixtures), or decreased mobility and arrest due to crosslink formation.^20–22^

In many chemical systems, chemical reactions are irreversible, leading to an arrest of the dynamics. Such arrest of kinetics can logically affect phase separation. For example, for conventional covalent polymer networks (*i.e.*, irreversible networks) that are undergoing crosslinking reactions, ongoing phase separation processes can become arrested, by effectively “freezing” the network topology into its concurrent state. It should be stressed that in this review we mainly focus on the phase separation of network components within the network. We stress that the arrest we discuss does not mean that a final crosslinked state is no longer able to phase separate. For example, at low crosslinking densities crystallisation might occur, or a crosslinked network may still react to stimuli such as temperature (*e.g.*, as in pNIPAM^23^) and induce heterogeneities and/phase separation between polymer network and solvent.

The incidence of arrest depends heavily on the crosslinking and solvent extraction kinetics in the polymer network, diffusion/reptation behaviour of the polymer strands, the presence of shear and magnitude of interactions between polymer segments.^20–22,24^ By influencing these parameters, the final phase-separated state of the polymeric network can be tuned. By stopping phase separation at specific times, materials with differently sized or structured domains can be formed. A recent review by Fernández-Rico *et al.* elegantly describes the state-of-the-art in liquid–liquid phase separation arrest in permanent polymeric networks.^20^

The partially completed phase separation that may result in nano-, micro- or macro-sized phases should be distinguished from the (equilibrium) microphase separation of well-defined block copolymers.^25^ The phase diagram of these systems may feature the well-known lamellae, hexagonal or cubic phases and, *e.g.*, more exotic phases such as the gyroid phase.^26–28^ These well organised (usually with long-range order) phases have in common that there is a molecular length scale similarly as in lipid bilayers, polymer or surfactant micelles.^29,30^ While block copolymers are a classical example, microphase separation is not limited only to block copolymers. Indeed, phase separation into graft-rich and graft-poor phases in graft copolymers is also frequently classified as microphase separation.^31^ The overarching characteristic for microphase separating systems is the fact that phase-separating components are chemically bound on the molecular scale. This constraint causes equilibrium macrophase separation of the components to be inhibited, and forces separation to occur on length scales of individual molecules.^32^ While the size of kinetically trapped domains may be of the same or similar length scale, and can be the result of nucleation and growth or spinodal decomposition processes, they typically lack the long-range order of the thermodynamically stable (microphase-)separated phases.

Next to arrested macrophase separation and microphase separation, it is also relevant to consider the crystallisation of polymer segments (driven by enthalpic and entropic contributions). In polymer networks, crystallisation is facilitated by the linearity and repetitive nature of polymer segments.^33^ By aligning in repetitive structures, such as lamellae, distinct domains are formed in the polymer network. Semi-crystalline polymer networks have been a subject of study for many years,^34–36^ and crystalline domains have been found to increase mechanical properties of the system. A good example of applying crystallisation for strengthening materials is found in thermoplastic elastomers (TPEs). Within TPEs, crystalline regions can function as physical crosslinks, providing elastic behaviour and strengthening, especially under extension.^37–40^ For example, Yang *et al.* showed that TPEs with crystalline end groups had a higher elastic recovery (90.8% instead of 69.3%) and higher tensile strength (0.71 ± 0.09 MPa instead of 0.52 ± 0.06 MPa) than similar TPEs with glassy end groups.^40^

Phase separation through crystallisation is characterised by differences in polymer organisation and order, or mechanical properties such as hardness and/or local density of the polymer. Furthermore, in block copolymers, the crystallisation might preferentially occur with a subset of the monomeric species or polymer segments. In this case, there is also a distinction in chemical composition between the crystalline and amorphous regions.^41,42^

### 1.2 Phase separation in reversible polymer networks

The development of polymer networks with reversible bonds has received increased attention in the last decades.^43–50^ We define reversible networks as networks containing either (1) supramolecular, non-covalent bonding motifs or (2) dynamic covalent bonding motifs. These bonding motifs provide reversibility by either freely dissociating and associating, or through bond exchange reactions. The resulting materials are of great interest due to their inherent reprocessability and possibility for self-healing.^51–54^ Through different stimuli (*e.g.*, heat or light), reversible polymer networks can be induced to flow and ease reprocessing. Additionally, the responsiveness of these adaptable materials enables potential applications in sensors or actuators.^55–59^

Importantly to reversible polymer networks, phase separation provides a way to create hybrid materials where different domains have different dynamic behaviour. A common expression of this concept is the creation of “hard” and “soft” phases in the material.^7,60–62^ Here, the hard and soft phases influence the possibility for crosslink shuffling and change material properties, such as toughness, through the individual qualities of the domains.

The dynamic nature of the bonding motifs makes reversible polymer networks uniquely interesting for applying phase separation. These linkages can facilitate segmental ordering,^63^ causing thermodynamically favourable phase separation. Furthermore, dynamic linkages may cause the polymer networks to not fully arrest, and facilitate phase separation after formation of a crosslinked network structure. When comparing this with polymer mixtures and the Flory–Huggins theory, in this case the solvent typically is less important compared to interactions between different polymer segments, due to expulsion of the solvent on network formation and generally spatially close network structure. This particular type of phase separation would occur if there is effective motion of polymer segments over time, due to diffusion of the crosslinks within the material. However, precisely this effective motion of polymer segments and crosslinks also raises further questions. For example, it is not immediately evident whether these dynamic materials behave more as a viscoelastic solid, viscous liquid or something in between. The range of behaviours possible through this motion is rich, and describing phase separation in such a system through classical liquid–liquid or solid–liquid phase separation may not be sufficient.

In this review, we aim to highlight the developments, potential, but also fundamental challenges of phase separation within reversible polymer networks. Specifically, phase-separated supramolecular and covalent adaptable networks (CANs) will be discussed. In Sections 2 and 3, we will aim to point out structure–property relationships, characteristic length scales and target applications for phase-separated supramolecular networks and CANs, respectively. In Section 4, we will identify current challenges and opportunities in applying phase separation in reversible polymer networks. Here, we especially focus on where the current framework of phase separation is lacking, and additional understanding is required to fully harness phase separation as a design tool in dynamic polymer networks.

### 1.3 Analysis and visualisation of phase separation

**Fig. 2:**
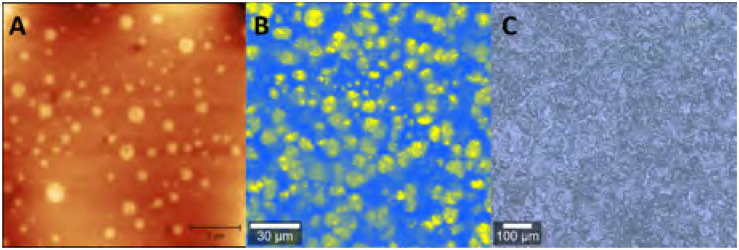
Example of (A) AFM, (B) Raman and (C) Brightfield images of phase-separated polyimine networks. Reprinted (adapted) with permission from ref. 66 Copyright 2022 American Chemical Society.

**Fig. 3:**
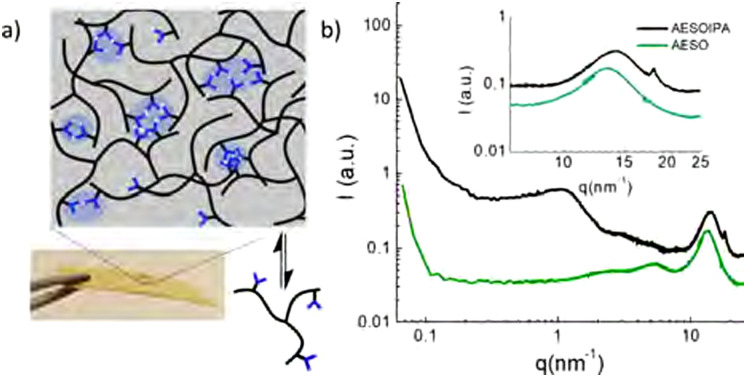
SAXS–WAXS spectra of acrylated epoxidised soybean oil and isophthalic acid (IPA) functionalised soybean oil. IPA-rich domains cause a diffraction peak around 1.5 nm^−1^ due to microphase separation, and a sharp band at 18.5 nm^−1^ suggesting π–π stacking of IPA motifs. Reprinted with permission from ref. 71 Copyright 2019 American Chemical Society.

Phase separation in polymers can be analysed and visualised using several different techniques.^64^ Phase-separated domains are commonly visualised by applying scanning electron microscopy (SEM), transmission electron microscopy (TEM) or atomic force microscopy (AFM),^65^ but other techniques such as Raman imaging, 3D multiphoton fluorescence lifetime microscopy or mechanofluorescent probes can be applied as well (Fig. 2).^7,66,67^ It is also seen more often that analysis methods are combined, such as the combination of AFM with FT-IR (AFM-IR)^68,69^ or AFM with tip-enhanced Raman spectroscopy (TERS).^70^ These techniques are able to show phase separation on the scale of nanometers to micrometers. Small angle X-ray scattering (SAXS) is another commonly used technique to study phase separation (Fig. 3), which can indirectly determine domain sizes based on a correlation function. To further analyse the effects of phase separation in terms of mechanical properties, differential scanning calorimetry (DSC) and rheology – often in the form of dynamic mechanical analysis (DMA) – are commonly applied. With both DSC and rheology the glass transition temperature (*T*_g_) can be determined, but in some cases, also multiple transition temperatures can be visualised. For example, when two (or more) distinct phase-separated domains vitrify or melt at different temperatures, a metastate between partial and full vitrification can exist, which can be observed in DSC by the occurrence of multiple thermal transitions.

## 2 Supramolecular polymer networks

### 2.1 Chemistry of supramolecular bonding motifs

Supramolecular polymer networks consist of monomers or polymer segments interconnected by supramolecular interactions.^48^ Major examples of supramolecular interactions are hydrogen bonding,^72^ electrostatic interactions,^73^ hydrophilic/hydrophobic association,^74–76^ or metal coordination,^77,78^ which can even be combined into multiphase-separated networks.^79^ However, other non-covalent interactions such as weak, non-directional van der Waals interactions or the fluorophobic effect can to some degree also be considered as weak dynamic crosslinks.^80^ Supramolecular networks are non-covalent and reversible in nature, and are often weaker than covalent bonds. However, through high connectivity and multivalence of the bonding motifs, strong networks can be formed.^81^

Hydrogen-bonding motifs typically remain the interactions of choice in organic supramolecular polymer networks due to their directionality, diverse association constants, and facile and wide-ranging options for incorporation.^82^ Their directionality and reliance on specific donors and acceptors allows for construction of regularly ordered complexes in materials. Furthermore, hydrogen bonds are diverse in bonding strength, and the number of hydrogen bonds in different motifs can strongly differ.^46^ This diversity has allowed for a wide variety of supramolecular systems,^60,71,83,84^ and have a profound impact on phase separation in dynamic networks.

Bonding motifs can be added in three different ways: (1) through end group functionalisation, (2) as side groups (or on branches) to the main chain, or (3) incorporated in the main chain. The location and number of bonding motifs present on polymer chains, blocks, grafts or branches have a profound impact on the behaviour of the supramolecular polymer network.^85,86^ In the context of phase separation, it especially affects the mobility and flexibility of strands, before and after incorporation in phases.

In the following paragraphs the effects of phase separation on supramolecular polymer materials will be discussed. Here, the primary focus will be on the effects of aggregation of bonding motifs, crystallisation behaviour and physical crosslinking. Through these physical concepts, the link between phase separation and material characteristics is established.

### 2.2 Aggregation and crystallisation of bonding motifs

Aggregation of bonding motifs is a widely used principle to add physical crosslinking behaviour and increased mechanical properties to supramolecular networks. Frequently, a directional interaction is used to achieve stacking, of which the most prominent example is probably π–π stacking,^87–89^ followed by hydrogen bonding,^90^ provided the motifs contain both hydrogen bond donating and accepting moieties. Aggregation of bonding motifs provides further mechanical strength and network behaviour. However, it also tends to confine the bonding motifs to nanoscale hard domains, which might prevent beneficial qualities such as self-healing ability.^60,91^

**Fig. 4:**
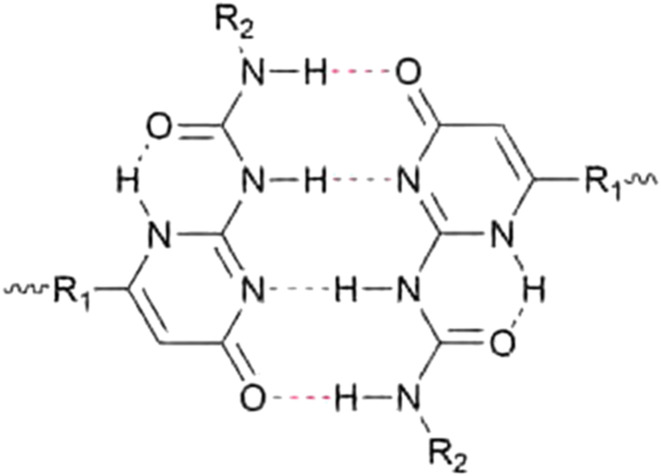
Chemical structure of a dimerised 2-ureido-4[1*H*]-pyrimidinone (UPy) motif through quadruple hydrogen bonding.

**Fig. 5:**
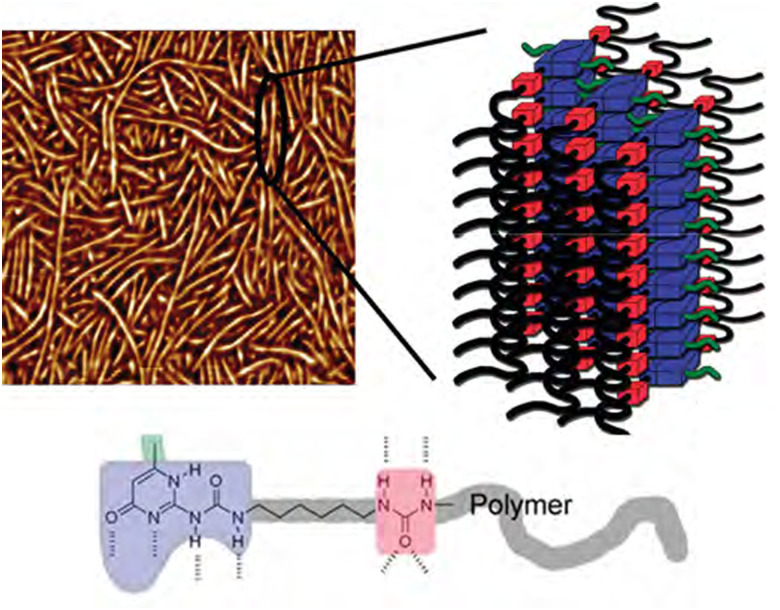
AFM image (500 × 500 nm) and schematic representation of nanofibers from UPy-rich polymers. Reprinted (adapted) with permission from ref. 96 Copyright 2011 American Chemical Society.

Because of the possible benefits of aggregation, the dynamics of aggregation have been studied in various systems and for different bonding motifs. One of the most widely employed bonding motifs that show aggregation and crystallisation behaviour is 2-ureido-4[1*H*]-pyrimidinone (UPy).^92,93^ UPy motifs have a quadruple hydrogen-bonding functionality, and readily undergo dimerisation to form strong complexes (Fig. 4).^83,94^ These complexes are highly specific, and allow extensive control over the supramolecular network properties. In addition to the high binding strength and directionality of the hydrogen bonding of UPys, π–π stacking of the pyrimidinone moiety allows for aggregation of UPy moieties into stacks (Fig. 5).^95,96^

UPy stacks are known specifically for their capability to crystallise, as well as their effective use as physical crosslinks (*vide infra*). This way, they can significantly improve the mechanical strength of polymeric materials. These beneficial qualities have led to extensive research into the application of UPy in supramolecular networks.^98–101^

**Fig. 6:**
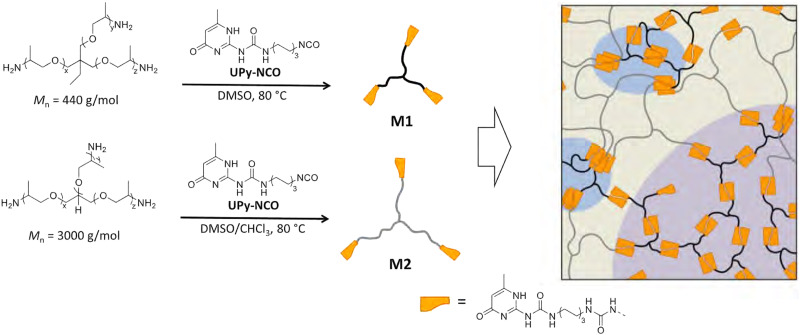
A schematic showing the three distinct phases formed through combining smaller (M1) and larger (M2) trifunctionalised poly(propylene oxide) monomers. The distinct phases are rich in (1) mainly M2 monomers (tan), (2) mainly M1 monomers (purple) or (3) crystalline UPy dimers (blue). Reprinted with permission from ref. 97 Copyright 2019 American Chemical Society.

For example, Hohl *et al.* showed how two trifunctional poly(propylene oxide) monomers of different sizes, end-functionalised with UPy, effectively formed three phases.^97^ These phases differed in the concentration of UPy dimers and aggregates. Increasing the relative contribution of UPy dimers and aggregates could tailor, and significantly improve, mechanical strength. This was achieved by increasing the relative concentration of monomer M1 to monomer M2 (see Fig. 6). For example, increasing the M1 : M2 content from 1 : 2 w/w to 2 : 1 w/w increased the Young's modulus threefold: from 81 ± 4 MPa to 245 ± 28 MPa. Similarly, Fan *et al.* showed that stacking of UPy dimers could effectively induce microphase separation, improving strength and toughness.^102^

Aggregation was also shown for other hydrogen-bonding and π–π stacking motifs. Del Prado *et al.* demonstrated that for isophthalic acid-functionalised triglycerides microphase separation and crystallisation of the isophthalic acid groups occurred.^71^ Additionally, SAXS results suggested the presence of π–π stacking within the hard isophthalic acid domains. Overall, these domains acted as physical crosslinks, strengthening the material.

**Fig. 7:**
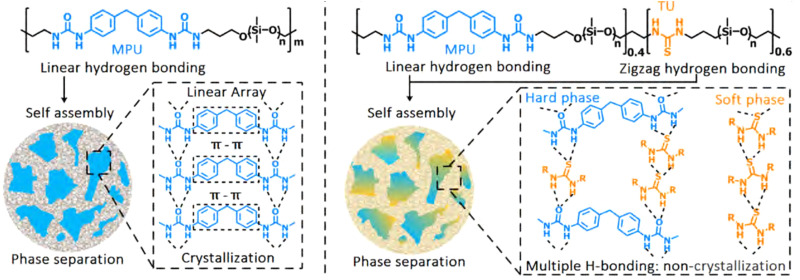
Stretchable and healable elastomers resulting from tuneable crystallisation behaviour between phases. Left: The original elastomer consists of hard crystalline phases incorporated in a soft matrix. The crystallinity of the domain caused lowering of the self-healing activity and stretchability of the final material. Right: A material with improved self-healing and stretchability, due to the presence of thiourea moieties in the backbone. These thiourea moieties disrupted the crystalline structure of the hard phase, as well as provided (weaker) hydrogen bonding in the soft phase, which caused improvement in material properties. Reprinted (adapted) with permission from ref. 60 Copyright 2019 American Chemical Society.

Since aggregation of bonding motifs increases mechanical strength, yet decreases self-healing ability, various efforts were made to find ideal structure–property relationships for specific aggregating moieties. For example, Xu *et al.* tuned the crystallisation behaviour of hard 4,4′-methylenebis(phenyl urea) (MPU)-rich domains in poly(dimethylsiloxane) (PDMS) polymers through variation in the relative thiourea (TU) content in the soft domains of the polymer (Fig. 7).^60^ Here, the TU groups positioned in between the MPU moieties, thereby preventing effective π–π stacking and crystallisation. This led to prevention of crystallisation and an effective increase in stretching ability (from 954% strain-at-break without TU, to strain without fracture of up to 31 500% with TU) and self-healing ability. In another study, Salimi *et al.* found that using low molecular weight additives in supramolecular polyurethanes with end caps (analogous to the MPU functionality) could reinforce the material, *e.g.*, increasing Young's modulus and ultimate tensile strength by 840% and 230%, respectively, upon adding 8 wt% of additive.^91^ Specifically, these additives mirrored the structure of the end caps of the polymer chains, thereby facilitating clustering behaviour and alteration of the phase separation.

**Fig. 8:**
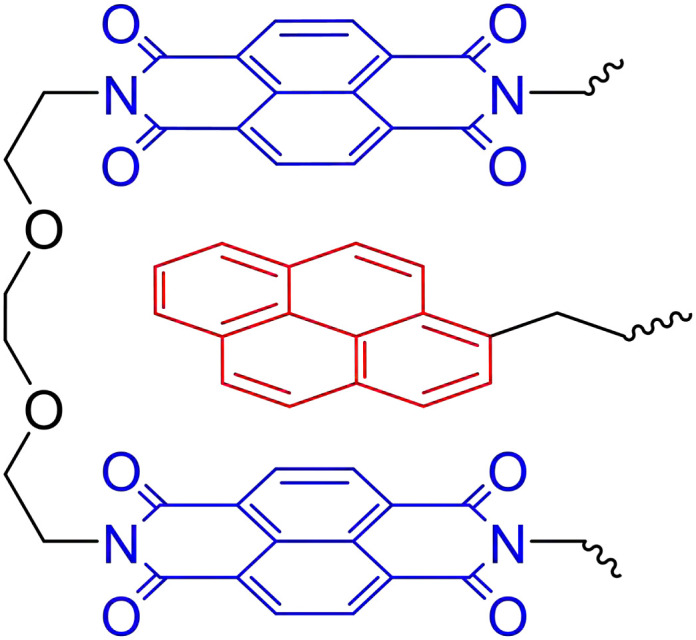
Complementary π–π stacking motif of π-electron-rich pyrenyls (red) and π-electron-deficient naphthalene diimide (blue).

π–π Stacking of similar (aromatic) moieties is often noticed in either materials or solutions, but stronger π–π interactions can be obtained when combining different π-electron-rich and π-electron-deficient groups.^87,103,104^ Such complementary π–π stacking interactions have also been described to construct strong supramolecular crosslinked polymer networks. In an example, Hart *et al.* used the combination of π-electron-rich pyrenyl groups and π-electron-deficient naphthalene diimide units (Fig. 8).^105^ To construct supramolecular polymer networks, pyrenyl groups were placed at the terminals of bi- and trivalent PEG polymers, which were then blended with a chain-folding copolymer bearing the naphthalene diimide groups. Assembly into supramolecular networks occurred rapidly in both solution and solid state when mixing the different polymers. Analysis of the formed material by SAXS revealed that phase separation had occurred with domain spacing of *ca.* 100 Å. They furthermore observed that, while the domain spacing was invariable within the studied temperature range, the X-ray contrast between the different domains increased with rising temperatures. This observation of scattering intensity as a function of the temperature illustrates the reversible nature of the phase separation.

#### 2.2.1 Induction of main chain order

Contrary to aggregation of the dynamic bonding motifs, main chain crystallisation adds mechanical strength through changing polymer chain mobility and interactions between strands. Crystallisation of polymer segments often occurs on longer length scales since larger fractions of the polymer are included. Recently, the interplay of bonding motif and main chain crystallisation has also gained more attention.

For example, Lacombe *et al.* showed that crystallisation of thymine motifs could lead to pre-ordering of polymer segments, and thereby facilitate organisation of the main chain in a lamellar phase.^84^ Furthermore, the presence of amides neighbouring the thymine motifs was shown to facilitate the crystallisation of thymine, while changing the amides to non-hydrogen-bonding ester groups resulted in loss of the crystallinity and only led to clusters being formed. Therefore, pre-ordering of polymers through thymines, supported by directional hydrogen-bonding motifs, was necessary for mesoscale ordering. The order–disorder transition of thymine-functionalised poly(propylene oxide) was studied by Cortese *et al.* earlier as well, finding that the mechanical properties were significantly improved due to the crystallisation and ordering behaviour.^106^ Specifically, the materials showed a sharp transition from a viscous liquid to a viscoelastic solid from 70 °C to 60 °C.

The influence of molecular architecture, bonding motif, and processing conditions on ordering of a crystallisable block have been studied as well. For instance, Hohl *et al.* systematically investigated how the crystallinity of a poly(butylene adipate) (PBA) core changed when it was end-functionalised with either UPy or 2,6-bis(1′-methylbenzimidazolyl)pyridine (Mebip), under different processing conditions.^107^ Here, UPy-functionalised polymers reached supramolecular structures through hydrogen-bonding, while Mebip-functionalised polymers were coordinated by adding Zn(NTf_2_)_2_. It was found that under solution-cast and compression-molded processing conditions, UPy phase separation and consequent crystallisation effectively stifled crystallisation of the PBA core. Contrarily, Mebip-functionalised polymer lacked phase separation, and all processing conditions yielded crystallisation of the PBA core. This shows the complex interplay of bonding motifs and main chain, and the possibility for material tuning through morphology and kinetic trapping.

In a different approach, Lamers *et al.* changed the architecture of a crystallisable oligodimethylsiloxane (oDMS) block within UPy-functionalised polymers.^108^ Through changing the block to be either (1) incorporated in the main chain or (2) added as pendant grafts, different polymer architectures were reached, with significant impact on the nanoscale morphology. Specifically, the pendant graft architecture led to long-range lamellar organisation, which ultimately led to a highly brittle material. In contrast, the linear, main chain oDMS showed competition between crystallised, phase-separated and amorphous states, leading to less crystalline lamellar domains. In comparison with the pendant graft architecture, this formed a more ductile material. In this way, the dependence of mesoscale crystallisation on molecular structure was demonstrated.

### 2.3 Phase separation through incompatibility

Aside from phase separation induced by aggregation and crystallisation of bonding motifs, phase separation can also be induced by incompatibility of polymer backbones or functional groups.

A nice example of phase separation as a result of incompatible moieties was documented by Guan and co-workers,^109^ who described hydrogen-bonding brush polymers that self-assembled into a two-phase system with hard domains from the polystyrene (PS) backbone and soft domains from the polyacrylate amide brushes. This work presented a material that was able to self-heal *via* reversible hydrogen bonding, but also showed superior strength due to phase separation of hard domains. Later, the same group documented on dynamic block copolymer materials that phase separated as a result of incompatible PS and poly(*n*-butyl acrylate) (PBA) blocks.^110^ PBA-*b*-PS diblock copolymers were synthesised, and end capped with dynamic UPy moieties. As discussed before, one would expect the UPy moieties to phase separate as well. However, it was possible to avoid aggregation of the UPys by adapting the UPy design *via* introduction of a 2,6-diisopropylphenyl group to disrupt the π–π stacking.^111^ This way, the phase separation was purely caused by the incompatible segments of the block copolymers. The dynamic UPy moieties could be placed within the soft phase of the material, where the higher chain mobility facilitated efficient reversible hydrogen bonding in the solid state.

**Fig. 9:**
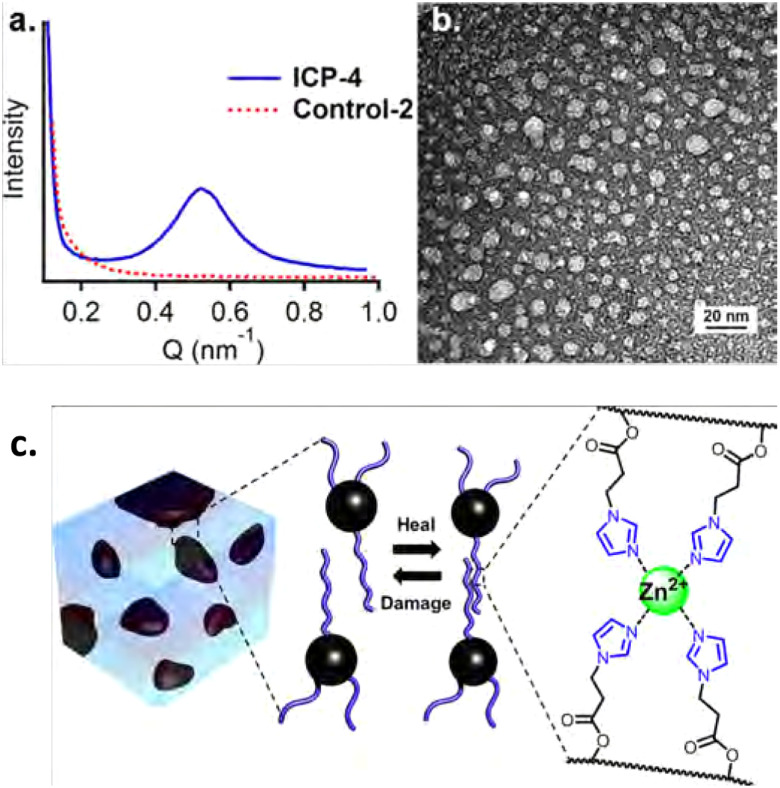
(a) SAXS and (b) TEM results, showing phase separation for (c) Zn^2+^–imidazole crosslinked polymers. Reprinted with permission from ref. 112 Copyright 2014 American Chemical Society.

Following the works by Guan and co-workers,^109,110^ the principles of the phase separation of brush polymers could be further applied to dynamic metal–ligand crosslinked polymer networks. In a later study by Guan and co-workers,^112^ a glassy PS backbone was used, on which imidazole-containing brushes were installed. When Zn^2+^ salts were added, the chains were crosslinked *via* complexation of the imidazoles with the Zn^2+^ ions (Fig. 9). The hard PS backbone phase separated again from the soft brushes, but this time the soft phase also contained the crosslinking Zn^2+^ complexes. As a result, a soft crosslinked matrix was formed in which hard PS domains reside. This way, a strong self-healing material could be prepared that was able to heal in the solid state with minimal intervention.

**Fig. 10:**
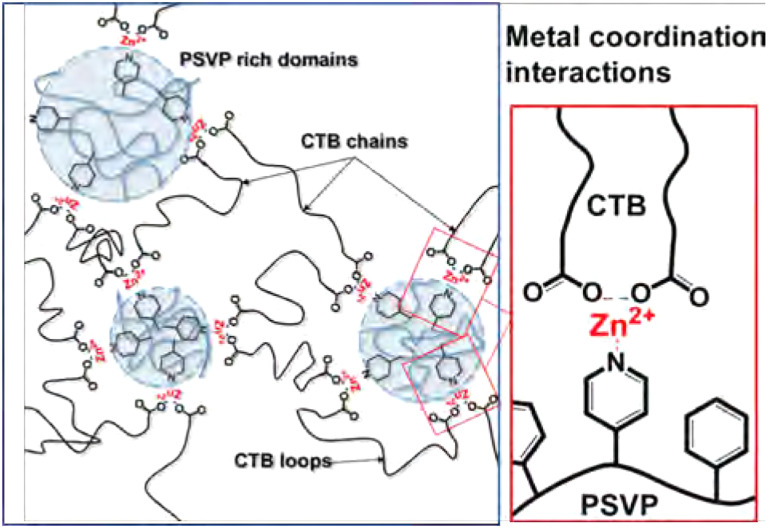
Visual representation of phase-separated metallo-supramolecular polymer network by Xie *et al.* Reprinted (adapted) with permission from ref. 116 Copyright 2016 American Chemical Society.

Phase separation has been observed for many other types of metallo-supramolecular materials as well.^113–115^ As an example, in the work by Xie *et al.*, supramolecular elastomers were constructed from a carboxyl-terminated polybutadiene (CTB) and poly(styrene-*co*-vinylpyridine) (PSVP), which were crosslinked by coordination of Zn^2+^ ions between carboxylic acid and pyridine moieties (Fig. 10).^116^ They found that microstructures of phase-separated PSVP domains dispersed in a polybutadiene-rich continuous phase. The phase-separated PSVP domains were, however, physically connected to the polybutadiene matrix *via* metal coordination of the pyridine groups of the PSVP with the carboxylic acid groups of the polybutadiene. These results showed that the two phases are physically connected *via* metal coordination, although technically the material would act as a thermoplastic blend between linear polymers (much like the phase separation observed in block copolymers). This physical crosslinking of hard and soft domains resulted in enhanced material strength, as the two phases were held together tightly. Specifically, 1 : 1 blends of CTB and PSVP enriched with Zn^2+^ ions had an increase in storage and loss modulus of roughly two orders of magnitude, compared to blends without Zn^2+^ ions.^116^

**Fig. 11:**
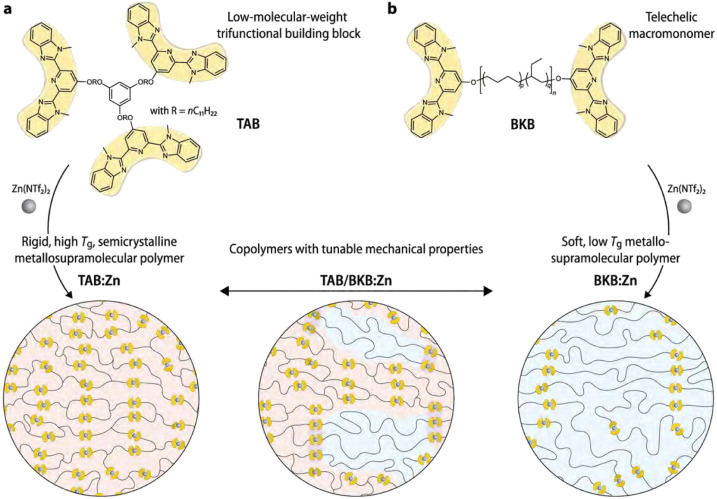
Visual representation of (phase-separated) metallo-supramolecular polymer network based on Mebip ligands and Zn^2+^ ions. Low-molecular weight trifunctional monomers create a rigid network or phases and high-molecular-weight bifunctional monomers make up linear soft phases. Only the combination of bi- and trifunctional monomers result in phase separation. Adjusting the ratio between the different monomers enabled tuneability of material properties. Reproduced with permission from ref. 61 Copyright 2022 Nature.

A different approach to enhance the material properties of metallo-supramolecular networks *via* phase separation was studied by Sautaux and co-workers.^61^ They looked into monomers with 2,6-bis(1′-methylbenzimidazolyl)pyridine (Mebip) ligand functionalities, which were placed on the termini of a rigid trifunctional monomer and a long, flexible bifunctional monomer. The monomers were then linked *via* metal coordination to Zn^2+^ ions (Fig. 11). When only the rigid trifunctional monomer was used for the creation of the supramolecular network, a tough and brittle semicrystalline network was constructed. However, when mixing the flexible bifunctional monomer into the material, a phase-separated microstructure was observed. Varying the ratio between the two components enabled tuneability of the material properties by increasing either the concentration of hard or soft phases. Furthermore, the synergistic effects of the phase-separated domains enabled significantly enhanced material properties, which pushed the limits of conventional metallo-supramolecular networks.

In other works on Mebip-type supramolecular metal-coordination networks, Neumann *et al.* started from an apolar linear bis-Mebip-functionalised macromonomer, which they linked *via* metal coordination to either di- or trivalent metal ions.^78^ A clear phase separation occured between the polar M–L complexes and the apolar polymer chains. Furthermore, they concluded that the microstructure and mechanical properties of the materials were significantly affected by the coordination geometry of the M–L complexes, as well as the volume fraction of the M–L phase. As a main trend, it was observed that for lanthanoid salts (forming weaker, more dynamic complexes), the softening temperatures significantly decreased, which were also observed by a loss of structural order. In addition, whereas lanthanoid materials displayed both hexagonal and lamellar microphase-separated structures, the transition metal materials selectively adopted lamellar configurations, irrespective of the metal ion, counter ion or polymer molecular weight.

## 3 Covalent adaptable networks (CANs)

Covalent adaptable networks (CANs) – sometimes also referred to as dynamic covalent (polymer) networks (DCNs or DCPNs) – consist of a covalently crosslinked polymer matrix with inclusion of dynamic covalent bonds.^117,118^ Dynamic covalent bonds can interchange through bond exchange reactions (BERs),^119^ which effectively transfer crosslinks throughout the material. BERs are often triggered by (mild) stimuli, *e.g.*, through heat, light or pH.^120^ This transfer of crosslinks throughout the material provides reversibility to the overall network, resulting in beneficial qualities such as self-healing ability and reprocessability.^121^ In this way, reversibility is added while the stability and strength of a covalent polymer network (thermoset) is preserved. Because of these promising qualities, recent research has focused on tuning BERs through molecular architecture and chemistry.^50,122–124^

### 3.1 Chemistry of CANs

Since the introduction of dynamic covalent (DC) bonds in crosslinked polymers, many different types of BERs have been investigated.^125–127^ Some prominent examples include transesterification,^128,129^ disulfide exchange,^130,131^ Diels–Alder reactions,^132,133^ (vinylogous) urethane exchange,^134,135^ dioxaborolane metathesis,^136,137^ and imine exchange.^138,139^ Several reviews have provided a more comprehensive overview of the different types of chemistries.^117,118,121,140–142^ An important distinction in these BERs is whether they occur associatively or dissociatively, *i.e.*, whether the reaction is (1) initiated with complexation of multiple DC motifs or (2) initiated with dissociation of a DC motif, after which a new DC bond is formed. The crucial distinction between these two behaviours is that in associative BERs the network integrity is maintained, while in dissociative BERs the network integrity is partly or completely lost during the reaction.^143^ Depending on the timescale of the dissociation and formation of DC bonds in dissociative CANs, they can however appear and behave similar to associative CANs when the equilibrium constant is high enough.^144^ In addition, for many dissociative CANs only a small percentage of dynamic crosslinks is broken instantaneously during reprocessing conditions, which limits the loss of network connectivity. Validation of either constant or altered crosslinking density upon heating is typically done by performance of frequency sweep experiments in rheology.^145^ In literature, associative CANs are commonly referred to as vitrimers,^141^ as coined by Leibler and co-workers.^146^ Note that fast-exchanging dissociative CANs are sometimes referred to as vitrimer-like.^142,147^

A major challenge in the field of CANs is joining robust material qualities with network reversibility at mild conditions. An especially well-known problem is the occurrence of creep in CANs at ambient conditions.^148^ Because of the (constant) bond exchange in CANs, they relax stress over time. As such, when a force is applied that leads to deformation of a CAN material, stress relaxation *via* bond exchange will compensate for this deformation, leading to creep of the material.^149^ To relieve the issue of creep in CANs, research has been focused on changing the reactivity and characteristics of the DC bonds, to tune the structural integrity and strength in the final materials. This has been done chemically, *e.g.*, by changing local polarity^150^ or sterics^144,151^ near the DC bonds, by means of metal-coordination of dynamic covalent groups,^152,153^ or by modifying the electronic behaviour of the DC bond.^154–156^ In addition, network characteristics have also been considered, such as the crosslinking density,^157,158^ or building in (a small degree of) permanent non-dynamic crosslinks.^159,160^

Recently, however, the possibility of tuning CANs through physical means has been proposed,^161–163^ making use of phase separation and aggregation, which will be discussed in the next paragraphs. By inducing phase separation in CANs, the aim is mostly to enhance mechanical and physical properties (*e.g.*, to increase the *T*_g_ or reduce creep), add new functionalities, and/or change the overall morphology of the material through dividing the material in different domains.

### 3.2 Phase separation in CANs

Phase separation in CANs has the potential to efficiently create self-assembled structures in materials,^164,165^ and enhance material properties.^166^ In this way, it can compartmentalise dynamic covalent bonds, as well as create materials that *in situ* behave as composite materials. This potential is especially relevant for relieving complex trade-offs in CANs between desired processability at mild temperatures and undesired creep or flow behaviour, as was mentioned in the previous section.

In the following sections, the most promising trends and results for phase separation in CANs will be addressed and discussed. In addition, we will address many of the underlying mechanisms behind phase separation, and how they can be exploited to construct well-defined materials with high-quality material properties.

#### 3.2.1 Aggregation-induced phase separation

**Fig. 12:**
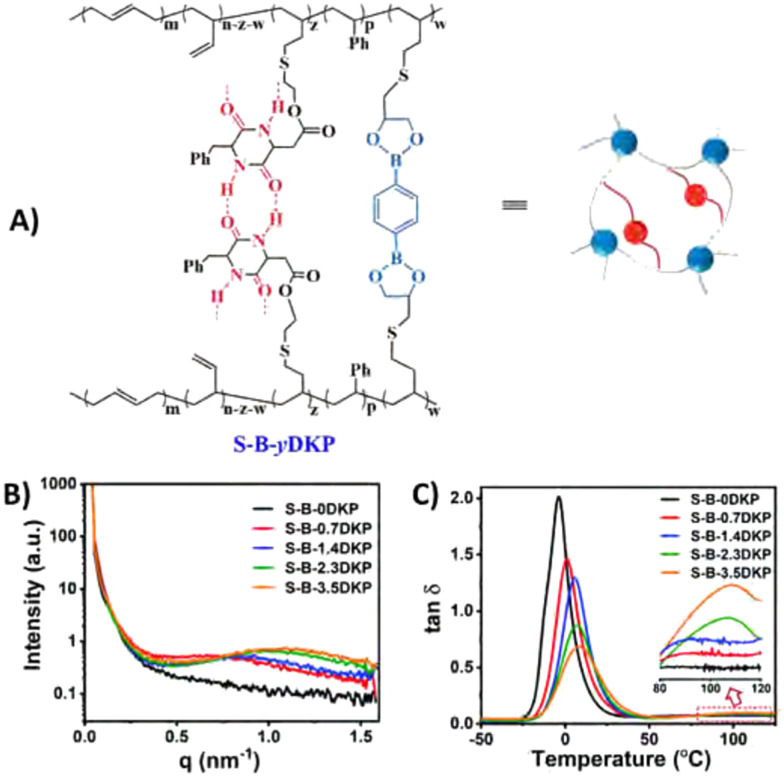
(A) Structure of dual-dynamically crosslinked styrene-butadiene rubber with boronic ester and hydrogen bonding motifs. (B) SAXS results, showing broad scattering peaks for the materials with hydrogen bonding motifs, indicating formation of microphase-separated domains in the rubber matrix. (C) tan(*δ*) curves, showing a gradual increase in *T*_g_ for higher hydrogen bonding concentrations, and a second *T*_g_ for materials with high hydrogen bonding concentration. Reprinted (adapted) from ref. 168 Copyright 2022 Royal Society of Chemistry.

Phase separation behaviour in CANs can be induced through addition of aggregating moieties in the polymer strands, as was seen before for supramolecular networks. An example of such phase separation *via* aggregation in CANs was given by Liu *et al.*, who reinforced a boronic ester-crosslinked vitrimer by introducing Zn^2+^–imidazole complexes.^167^ These complexes were found to aggregate upon addition of Zn^2+^ and form microphase-separated domains in the network. The presence of these phase-separated Zn^2+^–imidazole complexes within the material functioned as additional reversible units and were found to increase the strength, modulus, toughness and resistance to creep of the materials, while maintaining the malleability. In a similar fashion, Wang *et al.* reinforced boronic ester-crosslinked styrene-butadiene rubber by incorporating quadruply hydrogen-bonding moieties (Fig. 12(A)).^168^ SAXS confirmed that only when the hydrogen bonding motifs were installed, phase separation was observed (Fig. 12(B)). Rheological measurements of the materials then showed that the *T*_g_ increased gradually when increasing the concentration of the hydrogen bonding motifs (Fig. 12(C)). In addition, a second *T*_g_ was observed at 105 °C for the phase-separated materials, indicating dissociation of the hydrogen bonds. Creep experiments also showed that increasing the concentration of hydrogen bonding motifs was able reduce the degree of creep up to 10 times. The study explained this significant creep reduction as a result of the hydrogen bonding in the microphase-separated structure.

**Fig. 13:**
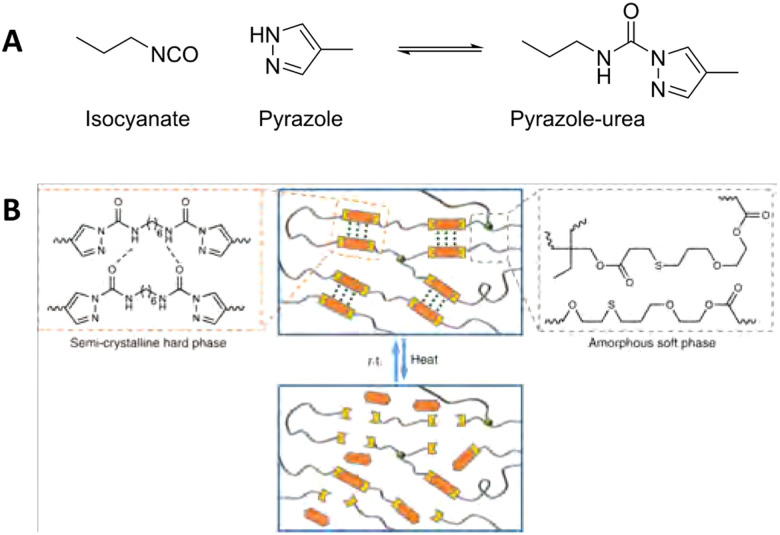
(A) Formation and equilibrium reaction of the pyrazole–urea from isocyanate and pyrazole. (B) Schematic representation of dynamic pyrazole–urea networks, in which the dynamic pyrazole–ureas motifs aggregate *via* hydrogen bonding. By heating the material, the polymer network breaks open, which enables reprocessability. Reprinted (adapted) with permission from ref. 170 Copyright 2019 Nature.

Phase separation induced by other hydrogen-bonding moieties are seen more often in CANs.^168,169^ Some specific dynamic covalent groups are also known to form hydrogen bonds themselves. Dynamic covalent pyrazole-urea groups are, for example, known for their reversible BERs, but also for their potential to form intermolecular hydrogen bonds. When incorporated into a polymer network, the intermolecular hydrogen bonding can thus cause aggregation of the pyrazole-urea moieties (Fig. 13).^170^ This way, the dynamic covalent groups are placed within close proximity of each other, which makes exchange between different polymer chains more efficient.

In recent work by Sun *et al.*, they exploited the hydrogen bonding induced aggregation of pyrazole–ureas to create phase-separated PDMS-based elastomers for application in laser sintering-based 3D printing.^171^ Analysis of the materials with SAXS clearly showed evidence for the formation of hard pyrazole-urea segments and a soft PDMS matrix. By comparing the SAXS results of the elastomers to linear polymers they could also conclude that phase separation only occurred for the network stuctures, and thus that the crosslinking was essential to get phase separation. The combination of dynamic covalent and supramolecular crosslinking, and the induced phase-separated structure resulted in good material strength with Young's moduli up to 21 MPa and a *T*_m_ of the hard domains up to 138 °C. They did, however, observe that introduction of large steric groups on the pyrazole moieties resulted in decreased material properties. Specifically, the tensile strength decreased from 10.4 MPa to 7.9 MPa and 7.1 MPa when introducing methyl or *tert*-butyl substituents on the pyrazole ring, respectively. It was also observed that the size of the hard domains increased from 8.5 nm to 9.5 nm and 10.5 nm for hydrogen, methyl and *tert*-butyl substitutions, respectively. They assumed that large steric groups cause loose arrangement of hard segments, causing easier slipping of molecular chains along the stretching direction under external force.

**Fig. 14:**
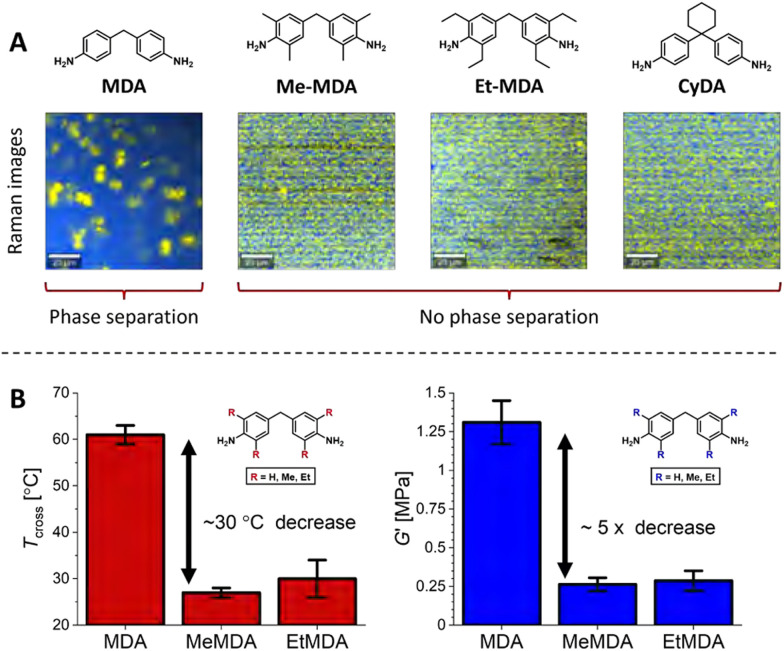
(A) Raman images of polyimine CANs synthesised from MDA monomers with different sterically demanding groups, (B) *T*_cross_ (where tan(*δ*) = 1) and *G*′ of the materials with either MDA (R = H), MeMDA (R = Me) or EtMDA (R = Et) as dianiline monomers. Reprinted (adapted) with permission from ref. 66 Copyright 2022 American Chemical Society.

Suppression of phase separation by steric effects appears to be a more common theme, as it was also observed in UPy-based supramolecular networks, for which aggregation and microphase separation disappeared when increasing steric bulk on pyrimidone moieties.^96,172^ In our own studies, we also observed that added sterically demanding groups on phase-separating dianiline moieties in polyimine CANs disrupted the phase separation (Fig. 14(A)).^66^ By making use of Raman confocal microscopy, we observed that polyimines synthesised from the non-sterically bulked 4,4′-methylenedianiline (MDA) showed phase separation into domains with sizes up till approximately 10 micrometer. However, when sterically demanding groups were introduced on the dianilines (either on the aromatic rings or on the methylene bridge), the phase separation was lost. The probable cause for the loss of phase separation was that the sterically bulked groups prevent efficient stacking of the aromatic moieties. The loss of phase separation resulted in a noticeable change in material properties (Fig. 14). First, the elastic modulus (*G*′) dropped with a factor of roughly five, and second, the crossover temperature (*T*_cross_, where tan(*δ*) = 1) decreased roughly 30 °C. The *T*_cross_ represents the point at which the material transits from a viscoelastic solid to a viscoelastic liquid, and is thus an important parameter that defines the physical state of the material at a given temperature.

In a different example in which aromatic monomers were used, Torkelson and co-workers observed that xylylenediamine chain extenders caused phase separation in specific polymer networks containing dynamic covalent hydroxyurethane crosslinks.^173^ They also observed that when the dynamic covalent groups were able to interact with the polymer matrix *via* hydrogen bonding or polarity effects (*e.g.*, for poly(tetramethylene oxide)), no phase separation was observed. However, when the polymer matrix was incompatible to the dynamic covalent groups (*e.g.*, for PDMS) phase separation occurred.^174^ Phase separation caused by incompatibility of PDMS was also observed by Li and co-workers.^175^ In their work, they described the synthesis of poly(hydroxyl ether ester) networks, crosslinked *via* maleimide Diels–Alders linkages. In a first attempt, short maleimide linkers were included, which resulted in brittle materials. When exchanging (parts) of the crosslinks with longer and more flexible maleimide-functionalised PDMS linkers, they envisioned to enhance malleability of the materials. However, incompatibility of the PDMS with the main chains of the poly(hydroxyl ether ester) caused a phase-separated profile. This example thus shows that alterations to an existing material could transform an amorphous and homogeneous material into a phase-separated material, with altered material properties as a result. Generally, phase separation is induced purposely to enhance material properties. However, in some cases phase separation is unwanted because the amorphous and homogeneous composition is preferred.

Phase separation caused by incompatibility of dynamic covalent groups and non-dynamic bonds within the same polymer has also been discussed, for example in work by Song *et al.*^176^ They constructed hybrid polymer networks including both dynamic and non-dynamic crosslinks and tuned the ratio between normal covalent and dynamic covalent crosslinks. They observed that when the dynamic covalent crosslinks minimally took up 20 mol%, continuous phases of dynamic covalent groups were formed in the material that enabled reprocessability and self-healing behaviour. This work thus suggests that a minimal threshold concentration of dynamic covalent groups was required in order to induce phase separation, resulting in the typical dynamic behaviour of a CAN. In our own studies on the phase separation in polyimine CANs, we also observed certain threshold concentrations for aromatically linked dianiline monomers compared to aliphatically linked imines.^66^ Below this threshold concentration (generally between 10–20%, depending on the structure of the dianiline), no phase separation occurred, and the aromatic monomers blended in with the aliphatic matrix.

#### 3.2.2 Crystallisation-induced phase separation

The combination of dynamic covalent bonds and (semi-)crystalline or liquid crystalline phases equips CANs with unique features, such as efficient heat storage and release^177^ or temperature-dependent shape memory.^178^ The kinetics for crystallisation of polymers are typically affected either by the polymerisation kinetics of the monomers, or after polymerisation by the physical–chemical nature of the polymer chains. For CANs, however, the crystallisation kinetics can be largely influenced by the bond exchange as well.^179^ The introduction of dynamic covalent bonds in a network generates an additional timescale for crystallisation, in which small polymer strands can rearrange, rather than entire polymer chains. Such differences in crystallisation kinetics may also affect phase separation into domains with different crystallinity.

The incorporation of two different phases with individual crystallinity was nicely described by Song and co-workers, who showed the principle of creating shape memory as a result of a dual temperature response by constructing CANs containing poly(ε-caprolactone) and poly(ω-pentadecalactone) phases.^180^ Owing to the large difference in crystallisation temperature (∼60 °C), the different phases could be “melted” separately, activating the bond exchange in either phase separately. Heating to specific temperatures thus enabled the programming or resetting of the macroscopic structure, and cooling/heating cycles to adjusted temperatures could activate the shape memory within the material.

#### 3.2.3 Reactivity-induced phase separation

**Fig. 15:**
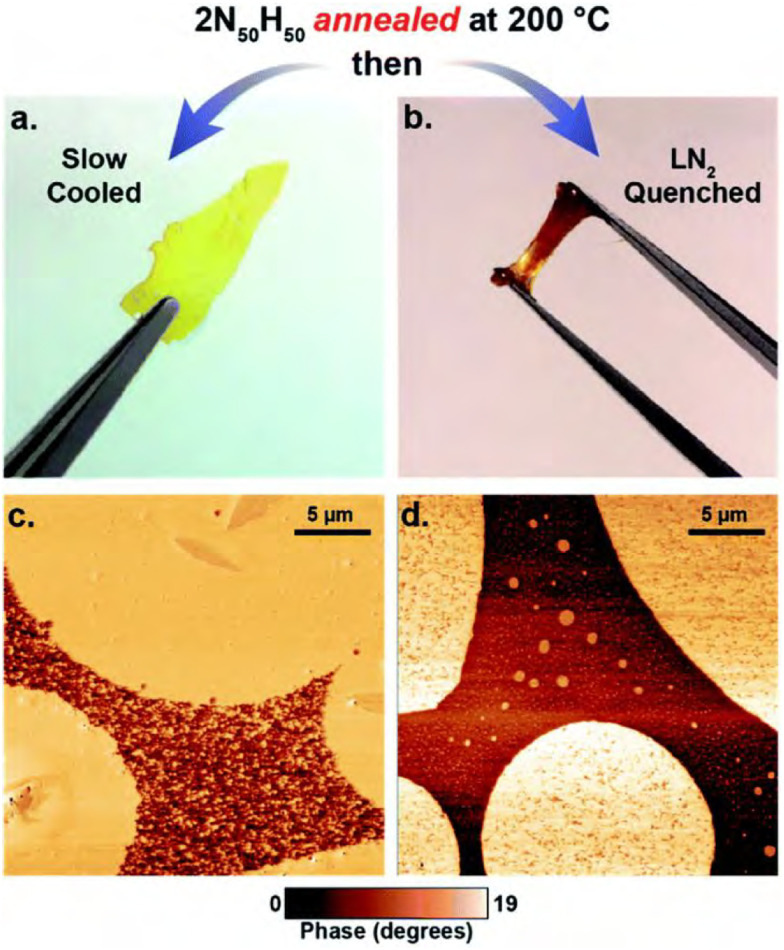
Appearance and morphology of the thia-Michael CANs with different annealing procedures. (a) and (b) show pictures of the materials after slow or rapid cooling, respectively. (c) and (d) show the corresponding AFM images of the microstructure of the materials. Reprinted with permission from ref. 181 Copyright 2020 Royal Society of Chemistry.

In work on thia-Michael CANs, Rowan and co-workers observed phase separation that appeared to be reaction-induced, and as such coined the term “dynamic reaction-induced phase separation” (DRIPS).^181^ They observed that the materials showed two distinct phase transition temperatures, which could be addressed to two different phase-separated domains. They further hypothesised that while dynamic covalent bonds are present in both phases, bond exchange only occurs between dynamic groups within the same phase. More interesting results were then obtained when they annealed the polymer films above the highest transition temperature, but varied the cooling time. They compared films that were annealed at 200 °C, after which one material was slowly cooled (1 °C per min.) and one was quenched in liquid nitrogen. The slow cooling resulted in the formation of a high percentage of hard phases that appeared continuously over the material, resulting in brittle glassy films. The fast cooling, however, disturbed the continuity of the hard phase, yielding a soft viscoelastic solid (Fig. 15). DRIPS thus offers an elegant way of tuning the material properties during reprocessing of the material without the requirement of additives to chemically transform the chemical composition of the polymer network.

**Fig. 16:**
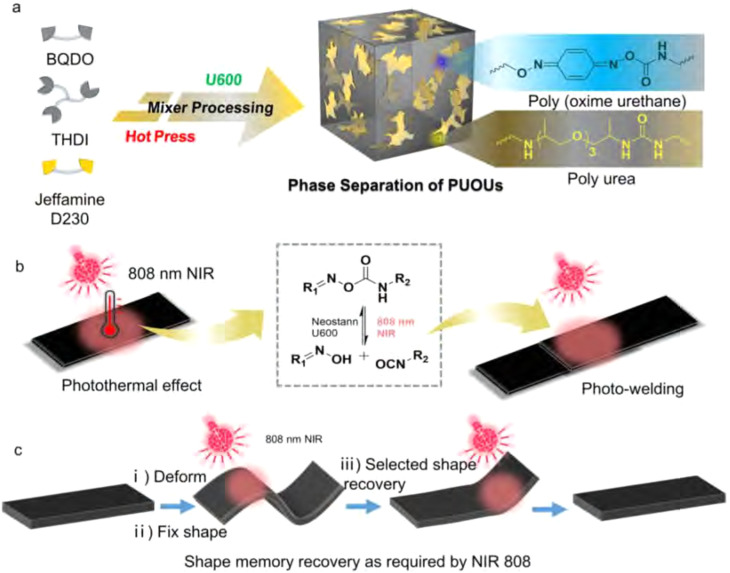
Schematic representation of (a) the formation of phase-separated poly(urea-oxime urethane) CANs, (b) NIR light-triggered photothermal activation of bond exchange, and (c) selective shape recovery effect of the materials. Reprinted with permission from ref. 182 Copyright 2022 Elsevier.

Chen *et al.* observed a similar dual phase transition with two individual glass transition temperatures for their poly(urea-oxime urethane) CANs (Fig. 16).^182^ They ascribed the phase separation as a result of different reactivity of monomers. In the synthesis, both an amine (Jeffamine D230) and an oxime (1,4-benzoquinone dioxime) were included to react with a trifunctional isocyanate monomer (Desmodur N-3300). The amine was found to react faster than the oxime,^183^ which resulted first in the formation of soft phases from reaction between amine and isocyanate, followed by formation of the hard phase *via* the slower reaction of oxime with isocyanate. Due to the two independent glass transition temperatures as a result of phase separation (Fig. 16(a)), and a NIR light trigger for bond exchange (Fig. 16(b)), selective shape memory behaviour could be facilitated and exploited (Fig. 16(c)).

#### 3.2.4 Fluorophobic effects

Replacing hydrogen with fluorine has a long history in polymer engineering, and can be used to affect phase separation by means of the fluorophobic effect. Although such fluorophobic effects are well studied for classical non-dynamic polymers,^184,185^ they are, so far, a less discussed topic in CANs. Fluorinated polymers and oligomers are specifically known for their unique properties, such as hydrophobicity, high thermal stability and solvent resistance, owing to the low surface energy of the fluorine atoms.^186^ However, when combining fluorinated and non-fluorinated chains into the same material, self-organisation and phase separation between the two phases will commonly occur. Such phase separation as a result of fluorophobic effects in fluorinated vinylogous urethane vitrimers was discussed by Du Prez and co-workers.^187^ They observed from DSC analysis that the fluorinated vitrimers displayed two distinct glass transition temperatures: one at −41 °C, which was assigned to the amine core and vinylogous urethane structure, and one at −100 °C, which was assigned to the fluorinated chains. Similar observations were made using DMA, confirming the existence of two separate phases. They also observed that the polymers with 5 mol% and 10 mol% of free amines in their structure slowly transformed from transparent to turbid samples within 3 days at room temperature. This observation suggested that the phase separation of fluorinated domains is a dynamic process, in which nanosized domains – that do not visibly scatter visible light – slowly aggregate into larger micron-sized domains that do scatter light.

### 3.3 Inclusion and phase separation of non-dynamic phases in CANs

Instead of constructing a material that has dynamic covalent bonds throughout its whole structure, a material might also benefit from a structure that only partly holds dynamic covalent bonds.^160^ A high concentration of dynamic covalent bonds may enable easier or faster reprocessing and self-healing, but may also show negative side effects, such as creep. It may thus be feasible to investigate ideal concentrations of dynamic covalent bonds compared to non-dynamic bonds.^159^ Such investigations were carried out by Rowan and co-workers, who studied the ideal ratio between dynamic alkylurea and non-dynamic urethane bonds in poly(alkylurea-*co*-urethane) networks.^188^ They observed that a minimal concentration of dynamic alkylureas of 50% (compared to non-dynamic urethane) was required in order to facilitate efficient recyclability. This work was initially focused on the use of non-phase-separated materials, although the authors did comment on the potential of phase separation. In other work by Rowan and co-workers they did discuss the entrapping of dynamic covalent disulfides within the hard phases of polyurethane networks.^189^ They observed that by trapping the dynamic covalent bonds in the hard phase, this served as a “switch-off” of the bond exchange below the melting point of the hard phase. In order to optimise the ratio between dynamic and non-dynamic crosslinks within a phase-separated material, Song *et al.* observed that relatively small amounts of non-dynamic crosslinks (less than 20 mol%) in hybrid disulfide-epoxy networks were already sufficient to significantly enhance the mechanical properties, without losing the reprocessing and self-healing capabilities.^176^

**Fig. 17:**
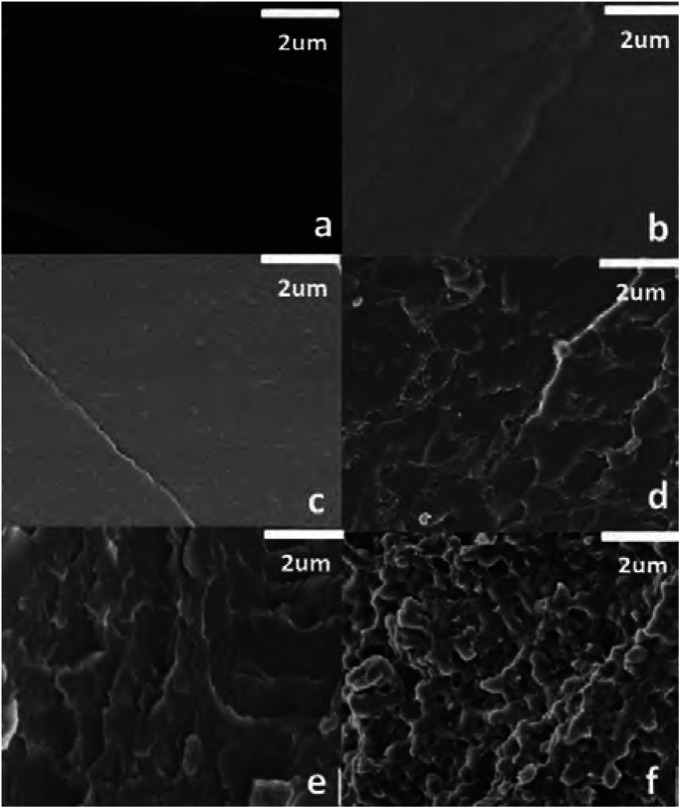
SEM images of PMMA–epoxy vitrimers with different PMMA contents. From a–f, the weight% of PMMA was 0, 5, 10, 15, 25 and 50%, respectively. Reprinted with permission from ref. 190 Copyright 2018 John Wiley and Sons.

Phase separation between dynamic (*i.e.*, phases with dynamic covalent bonds) and non-dynamic phases (*i.e.*, phases without dynamic covalent bonds) into CANs were also studied by Han *et al.*^190^ They constructed an epoxy vitrimer from epoxy resin and citric acid, which was doped with methyl methacrylate and benzoyl peroxide. The produced material showed either blending, entanglement, or phase separation of the poly(methyl methacrylate) (PMMA) domains from the vitrimer matrix based on the concentration of PMMA, as could be seen from SEM images (Fig. 17). Below 10 wt% PMMA no phase separation was observed and the components blended well within the material (Fig. 17(a)–(c)). Increasing the concentration of PMMA to 15–25% resulted in the formation of a PMMA network structure within the material (Fig. 17(d)–(e)). When an even higher PMMA concentration of 50% was used, a clear two-phase phase separation occurred (Fig. 17(f)). By increasing the concentration of PMMA, the *T*_g_ of the vitrimer material was noticeably affected. The materials with low PMMA content and without phase separation had a single *T*_g_ around 67 °C, similar to that of the pure epoxy vitrimer. However, for the phase-separated materials with higher PMMA content, a second *T*_g_ was observed around 100 °C. An interesting trend in the stress relaxation was also observed. When increasing the PMMA content from 0 to 25% the relaxation time decreased gradually from roughly 3 h (0% PMMA) till roughly 1 h (25% PMMA). However, for the two-phase phase-separated 50% PMMA material, the relaxation time significantly increased up to roughly 10 h. From this increase it was concluded that the PMMA promoted the transesterification reaction, resulting in a decrease in relaxation time, whereas the phase separation suppressed the overall stress relaxation.

### 3.4 Arrest of bond exchange through phase separation

Phase separation within dynamic covalent networks has the potential to effectively tune the kinetics and localisation of bond exchange reactions. A prominent example is the inclusion of dynamic covalent bonds within hard phases in a phase-separated network. Here, the bonds are inhibited from exchanging freely due to the rigid microstructure of the material. This concept has been coined “phase-locking” by Dong and co-workers, who used this concept to create self-healing elastomers with improved mechanical and healing properties.^62^ Specifically, these elastomers consisted of polyurethane polymers with hard and soft segments. The hard segments were strongly hydrogen-bonding and rich in dynamic covalent disulfide bonds, while the soft segments consisted of polytetramethylene ether glycol (PTMEG). Importantly, the hard segments were prevented from crystallising through steric hindrance provided by hydrogenated 4,4′-methylenediphenyl diisocyanate. Later, this phase-locking effect was also studied by Dong and co-workers for dynamic disulfide bonds embedded between two 4,4′-methylenediphenyl groups, to study π–π stacking-based phase-locking.^191^ This yielded hard segments that were crystalline and had a significantly higher melting temperature, and thereby caused the final material to have a high thermal resistance.

**Fig. 18:**
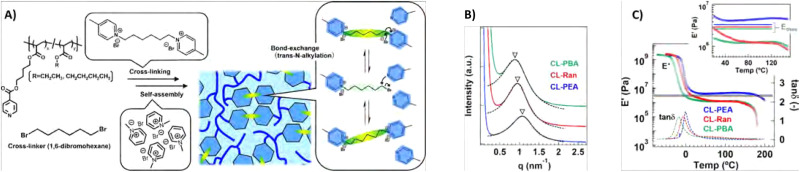
(A) Overview of the formation of *trans-N*-alkylatation-based polyacrylate CANs with either ethyl (CL-PEA), butyl (CL-PBA) or blended ethyl and butyl (CL-Ran) alkyl chains. (B) SAXS results of the CANs. (C) Temperature sweep results, showing tan(*δ*) and *E*′ curves. Reprinted (adapted) with permission from ref. 192 Copyright 2022 American Chemical Society.

In another example, Oba *et al.* observed phase separation in CANs based on polyacrylate-based random copolymers with dynamic crosslinks based on *trans-N*-alkylation of quaternised pyridine moieties (Fig. 18(A)).^192^ In these materials, self-assembly of the quaternised pyridine crosslinks *via* electrostatic associative interactions led to differences in network connectivity and relaxation of the material. An interesting correlation was then observed between the size of the aggregates and the bond exchange dynamics. First, it was observed that at higher temperatures larger aggregates were formed that showed faster bond exchange and stress relaxation due to close packing of the dynamic moieties. In addition, according to SAXS measurements, the size of the aggregates was also linked to the size of alkyl chains in the polymer structure, for which increasing alkyl chains led to larger aggregates (Fig. 18(B)). Not only the stress relaxation was affected by the increased bond exchange in the larger aggregates, but also the *T*_g_ and elastic modulus decreased in a similar fashion (Fig. 18(C)). As such, by controlling the aggregation size, the dynamic behaviour of the CAN could be tuned.

#### 3.4.1 Grafting of incompatible dynamic motifs for main-chain crosslinking

**Fig. 19:**
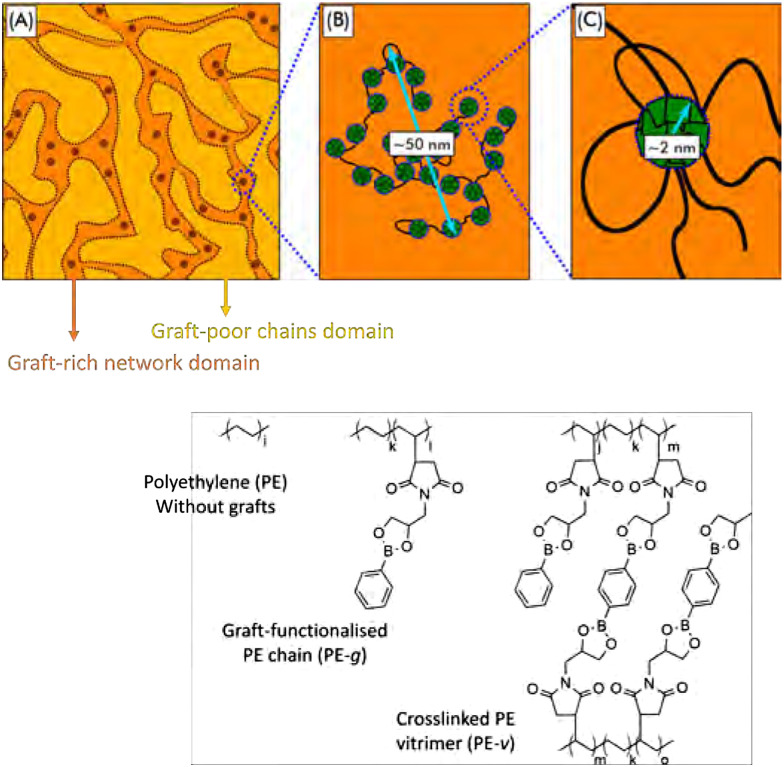
Schematic overview of phase-separated boronic ester grafted PE CANs into graft-rich and graft-poor domains. Reprinted (adapted) with permission from ref. 193. Copyright 2020 American Chemical Society.

A prominent example of phase separation in CANs was reported by Ricarte *et al.*, by graft-functionalisation of polyethylene with dioxaborolane maleimide grafts (Fig. 19).^193,194^ For these systems, it was shown that macrophase separation into graft-poor and graft-rich domains occurred. Furthermore, aggregation of dioxaborolane grafts took place on the nanoscale, likely due to the incompatibility of the dioxaborolane grafts with the polyethylene backbone. They found that this phase separation behaviour led to intricate meso- and nanostructures, which influenced the crystallinity and mechanical properties of the materials as well.^193^

In later work on the same system, the flow behaviour of these materials was studied. Here, the macrophase separation and aggregation caused by the graft functionalisation was found to decelerate the dynamics of the individual polymer chains.^194^ Furthermore, adding associative crosslinks, *i.e.*, bis-dioxaborolane, prevented the material from reaching terminal relaxation. Work by Nicolaÿ and co-workers on similar systems also showed that the degree of incompatibility of the grafts with the polyethylene strongly affected the nano- and macrostructure, dictating the thermal-mechanical properties of the material.^195^ Their work suggests that control over the degree of incompatible grafts could serve as a tool to regulate the nano- and macrostructure, and as a result tune the material properties. Peng and co-workers showed that this grafting principle could also be used to create leakage-proof and malleable polyethylene wax, by employing the phase separation into highly crosslinked and lesser crosslinked phases.^196^

#### 3.4.2 Dynamically crosslinked block copolymers

**Fig. 20:**
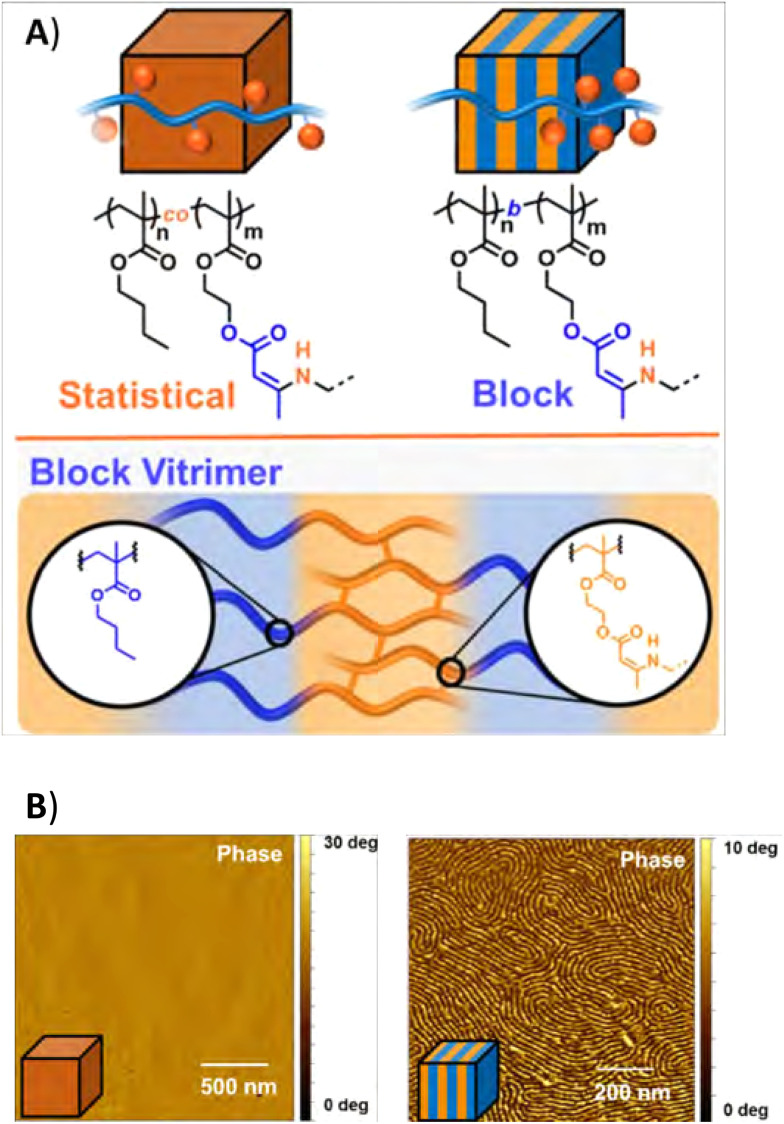
(A) Structure of statistical and block coplymer vitrimers, and (B) AFM images of the materials. Reprinted (adapted) with permission from ref. 197 Copyright 2020 American Chemical Society.

In a recent study, Sumerlin and co-workers used the polymer architecture to change morphology and phase separation by constructing block copolymer vitrimers (Fig. 20),^197^ as traditional linear non-dynamic block copolymers are known to be able to phase separate well.^25^ Specifically, the study by Sumerlin and co-workers focused on the difference between statistical and block copolymer vitrimers of the monomers butyl methacrylate (BMA) and 2-acetoacetoxyethyl methacrylate (AAEMA). Here, the AAEMA monomers were crosslinked by addition of xylylene diamine to make dynamic covalent vinylogous urethane crosslinks. Importantly, it was found that the block copolymer vitrimers, with a lamellar morphology on the mesoscale, exhibited significantly decreased creep and flow at longer timescales and large deformations. This was attributed to the microphase-separated structure, showing the potential of microphase separation for topology control and material properties in dynamic covalent networks. Similar conclusions were drawn in later works, in which significantly increased material properties were observed for phase-separated di- or triblock vitrimers as compared to the random polymers.^198,199^ The increased creep resistance and slower bond exchange (*i.e.*, higher activation energy) were mainly attributed to hindered polymer strand diffusion as a result of the phase separation. In other work by Chen and co-workers, they even pushed the limits of phase separation in dynamic block copolymers to construct crosslinked triblock copolymers that were able to self-assemble into interlinked, hexagonally packed cylinder nanostructures, limiting inter-cylinder bond exchange.^164^ By this means, creep could be significantly suppressed. Other work on dynamically crosslinked block copolymers by Kalow and co-workers showed that network connectivity defects, such as loops and dangling ends, are increased by microphase separation.^200^ As such, they speculate that the material properties of dynamic covalently crosslinked block copolymers arise from the interplay of network defects and phase separation.

## 4 Future challenges and opportunities

From all the mentioned examples of phase separation in dynamic polymer materials we infer that there is a growing interest in the studies towards phase separation. We have seen that material properties can be tuned and enhanced as a result of phase separation, and that more information is being uncovered regarding the mechanisms of phase separation. However, although great efforts have been made, many aspects of phase separation remain unclear. Several mechanisms for phase separation have been covered in this review (*e.g.*, *via* intermolecular interactions or incompatibility), but more underlying physical/chemical pathways that induce phase separation might exist. For example, in biological systems phase separation occurs on many levels.^201^ Nature can thus serve as an inspiring source for other modes of phase separation and compartmentalisation. Collaborative work from the fields of polymer chemistry and biochemistry could thus well go hand-in-hand as many biological macromolecules can be considered and modeled similar to synthetic polymer materials.^13^

To fully grasp the potential of phase separation in dynamic networks, increased fundamental understanding of the underlying processes, and their interactions with each other, is paramount. So far, modelling has been limited mostly to dynamic networks with a singular phase, although different chain arrangements (*e.g.*, random and block copolymer systems) have been discussed.^202^ These models perform adequately for explaining bond exchanges, mobility of crosslinks, self-healing and other processes inherent to dynamic networks.^203–212^ However, these models cannot be extended directly to phase-separating systems, since within these systems different processes start interacting. An example of this is the possible effect of local concentration differences on the mobility of polymer strands, accessibility of dynamic bonds and overall chemical environment. These local differences may have profound impact on mesoscale and macroscale properties of the material, such as temperature responsiveness, self-healing activity and mechanical performance. While it would be possible to model multiple phases separately, important information on the interfaces, mechanical response over longer length scales and formation of phases is lost. Therefore, significant advances in computational modelling are required to be able to explain the link between mesoscale structure and material properties.

A different complexity inherent to dynamic networks is the definition of phase separation arrest. Since the bonding motifs are dynamic under certain stimuli, or even at ambient conditions, phase separation does not face full arrest as in conventional, non-dynamic polymer networks. Therefore, arrest is not as clear-cut, and different contributions must be considered. For example, is it possible to continue phase separation in a fully cured network, by dynamic rearranging alone? Is it possible to temporarily arrest phase separation by removing the stimuli for bond exchanges? These possibilities and open questions make dynamic polymer networks unique candidates for application and harnessing of phase separation as a design tool. Some degree of control over phase separation during annealing was already shown,^181^ and further research could develop this into reversibly self-assembling structures within the material. We also observed in earlier work that phase separation in polyimine CANs was reversible, even after curing.^66^ When paired with functionalities such as reduction of creep and shape memory, this becomes a viable method for creating smart materials.

Other fundamental questions related to behaviour specific to CANs also remain, for example for the earlier discussed phase-locking.^62,191^ While the effects of phase-locking are clear within binary “hard” and “soft” phases, dynamic reactivity at the borders of the two phases has not been studied in detail. Such interfacial reactivity could have a profound impact on cohesion, adhesion and fracture mechanics of the composite material.

Next to this, the differences in phase separation behaviour between dissociative and associative CANs has been insufficiently explored. The potential effects of the dynamic covalent exchange mechanism should not be understated, since maintaining network integrity might hamper reaching a fully homogeneous state and full dissolution of the domains. In associative exchange, migration of subunits from a phase depends on mobility through bond exchanges, while in dissociative exchange domains dissolution of the crosslinks, might lead to mobile subunits capable of moving *via* reptation or (if sufficiently small) diffusion. Further studies on the different mechanisms that describe either associative or dissociative mechanisms are of high relevance, especially as previous studies has shown that some materials relying on dissociative exchange mechanisms can still show behaviour that is more typical to associative CANs.^213^

Another possible line of research would be the inclusion of stimuli-responsive behaviour in only one of the phases. An example of this could be dynamic exchange being triggered only under a certain threshold of UV light. If this were to occur within phase-separated domains that are embedded in a polymer matrix, the domains may be induced to re-dissolve into the matrix due to the heightened dynamic exchange. This, in turn, would give a stimuli-responsive control over the properties of the sample.

## 5 Conclusions

We have discussed the applications and modes of action of phase separation in dynamic polymer networks. We have seen that either in dynamic covalent or supramolecular crosslinked polymers, phase separation operates on many levels, and has a vast influence on the material properties. Throughout this review we mainly focused on the mechanical properties of the materials, such as the *T*_g_, creep resistance or dynamic moduli, but also other material properties may be affected (*e.g.*, the colour or conductivity). Many of the described studies concluded that phase separation has a desirable effect on the mechanical properties of the materials. Phase separation is clearly affected by heterogeneities in the chemical and physical composition of the material. These heterogeneities can originate in the different chemical nature of building blocks (*e.g.*, repulsive or attractive forces), differences in reactivity during the formation of the polymer, or different dynamic behaviour of functional groups within the formed network. Once the underlying mechanisms that cause phase separation are understood, materials can be tuned on the microscopic level in order to either induce or prevent phase separation, leading to control over the macroscopic material properties. With applications in mind, it is thus important to exploit the potentials of phase separation in the construction of advanced materials with novel functionalities.

## Author contributions

The topic of this review was jointly proposed by MdHK, SS, JD and MS, following earlier experimental work on phase separation in CANs. The collection of relevant papers fitting the topic was performed by MdHK and SS. All authors were involved in selection, analysis and categorising of the collected papers. The original draft of the manuscript was written by MdHK and SS. All authors contributed to revising and editing of the manuscript. Funding for this work was obtained by MS.

## Conflicts of interest

There are no conflicts of interest to declare.

## Supplementary Material
